# Physical activity levels among rural adolescents with a history of ankle sprain and chronic ankle instability

**DOI:** 10.1371/journal.pone.0216243

**Published:** 2019-04-30

**Authors:** Brittany Holland, Alan R. Needle, Rebecca A. Battista, Stephanie T. West, Richard W. Christiana

**Affiliations:** 1 Department of Kinesiology, School of Health and Human Sciences, University of North Carolina at Greensboro, Greensboro, North Carolina, United States of America; 2 Department of Health & Exercise Science, Beaver College of Health Sciences, Appalachian State University, Boone, North Carolina, United States of America; 3 Department of Recreation Management & Physical Education, Beaver College of Health Sciences, Appalachian State University, Boone, North Carolina, United States of America; Indiana University-Purdue University Indianapolis, UNITED STATES

## Abstract

Ankle sprains and their common sequalae are thought to negatively affect physical activity levels and health-related quality of life among active populations, but limited evidence has described this among younger populations. This study aimed to determine the prevalence rate of ankle sprain and chronic ankle instability among rural adolescents and subsequently compare their physical activity levels based on ankle injury status. The study was conducted in a rural high school in North Carolina. High school students completed an online survey that assessed ankle injury history, perception of ankle instability and function, and physical activity. Respondents were categorized into one of four groups based on ankle injury history and complaints of instability: 1) uninjured (no history of injury); 2) unstable (history of injury >1 year and recurrent instability); 3) copers (history of injury >1 year and no recurrent instability); and 4) potentially unstable (injury within the past year). Frequency of physical activity was compared across groups using analysis of variance, Kruskall-Wallis test (α = 0.05), and responses to activity type were assessed using chi-square. Physical activity was found to differ significantly between the four groups $\left(\chi_{4}^{2}=11.65,p<0.01,\eta_{p}^{2}=.07\right)$ with unstable respondents reporting more physical activity than uninjured respondents (unstable = 4706.05 ± 4610.56 MET-minutes/week; uninjured = 2592.93 ± 2946.02 MET-minutes/week). No differences were found between other groups. Despite injury history and sensations of instability, respondents with chronic ankle instability reported greater physical activity levels than uninjured participants. As this is contrary to pre-existing hypotheses, it is possible that continued physical activity after injury among adolescents may contribute to deleterious outcomes such as increased frequency of chronic instability.

## Introduction

Physical activity (PA) behaviors develop through sports and recreation during childhood and decline with age beginning in adolescence [1, 2]. This trend, which can lead to increased health risks, is particularly prevalent among girls [3]. Given the association between PA and decreased risk of all-cause mortality, heart disease, obesity, asthma, and certain types of cancer [4–6], it is important to be able to identify factors, such as musculoskeletal injuries, that may contribute to long-term decreases in PA. Persistent pain, sensations of joint instability, and decrements in strength and proprioceptive acuity have the potential to interfere with individuals’ desire to participate in PA and recreational activities [7, 8].

Ankle sprains are the most common injury among physically active individuals, with long-term sequelae extending beyond the initial injury, including repeated sensations of rolling of the ankle that, with repeated injury and perceived instability, contribute to chronic ankle instability (CAI) [9–11]. While incidence levels have been established among a range of active populations, limited evidence exists regarding the prevalence of these injuries in a variety of populations. Among college-aged individuals in the United States, and a large cohort of individuals in Australia, the prevalence of having had an ankle sprain at some point in their life has been reported near 60 percent [12, 13]. These values help to elucidate the economic burden of these pathologies, as ankle sprains and their sequelae have since been estimated to generate an economic burden of 4 to 6 billion dollars in the United States healthcare system [14]. Yet this value only estimates the initial treatment of these injuries. Given the potential of these injuries to negatively impact PA levels, this economic value may actually be greater than currently reported.

Limited research has investigated the effects of ankle injuries on PA levels among adolescents of high school age in the United States (generally 14–18 years old). However, a recent study using accelerometer-based step counting on college-aged individuals with CAI reported decreased PA levels compared to college students with no history of lower limb injury [15]. Adolescents are a population of interest because PA has been known to decline as individuals age [16, 17]. The impact of ankle sprains and CAI on adolescents’ PA levels has not been studied, and therefore leaves a gap in the research. Determining the prevalence of ankle injury and CAI and understanding the potential effects on adolescents’ PA levels may help to focus public health efforts on increasing awareness of the severity of ankle injuries and seeking proper treatment.

The purpose of the current study is to determine the prevalence of ankle sprains and ankle instability among adolescents in a rural county in North Carolina and to compare PA levels and activity participation across adolescents’ ankle status. It is hypothesized that adolescents who suffer from an ankle sprain engage in less PA than those that have not sustained a sprain. In addition, it is hypothesized that PA levels will decline as the degree of ankle instability increases.

## Methods

### Study design

A cross-sectional design was implemented in this study. Participant recruitment and data collection took place between November 2016 and January 2017. Dependent variables included self-reported measures of ankle function, PA levels, and activity type. Independent variables included ankle injury status.

### Measures

The online questionnaire was administered using Qualtrics software (Provo, UT). Included in the paginated questionnaire were questions regarding participant demographics, ankle injury history, and PA level. To determine the presence of CAI, the Identification of Functional Ankle Instability (IdFAI) instrument was used [18]. This instrument has demonstrated high reliability for the identification of ankle sprain and instability history (Cronbach’s alpha = 0.89–0.97). Self-reported ankle function was determined using the Foot & Ankle Ability Measure (FAAM) and the sport (FAAM-sport) subscale, which has been shown to reliably quantify individuals’ perceived ankle function (Cronbach’s alpha = 0.96–0.98) [19, 20]. Self-reported PA was quantified using the International Physical Activity Questionnaire (IPAQ) short-form, which has been used in similar studies [21] and recommended as a means to measure population prevalence as well as having shown similar criterion validity with accelerometry to other self-report measures for young adults with a Spearman’s *p* = 0.30 [22]. Finally, participant’s choice of recreational activities was determined using an adapted questionnaire from the United States Department of Agriculture Forest Service National Kids Survey [23]. The survey asked participants to select among 12 categories of types of recreational activities in which respondents participated during the past week and the limiting factors for participating in recreational activities. Categories included activities such as attending organized outdoor events such as camps, biking, jogging, hiking, swimming, playing with friends, motorized sports, team sports, and sedentary activities.

### Procedures

Potential participants were recruited directly from a high school in a U.S. Census Bureau classified rural county in western North Carolina. All study procedures were developed with the high school administration and approved by the Appalachian State University institutional review board. Parents had one full week to have their child return the signed passive consent form if they did not want their child to participate in the study. As all students are provided a laptop and email address by the school district, an email was sent to all potential participants with minor assent language and a link to the online survey. The survey took participants an average of about 12 minutes to complete.

### Data reduction

Out of 1,365 students, a total of 369 surveys (27.0%) were submitted from girls and boys 14–19 years old. Of the 369 surveys, 168 (45.5%) were discarded due to more than 60% of the data missing, resulting in 201 surveys being included in the analysis. From the remaining 201 surveys, participants were stratified into four groups using criteria from the International Ankle Consortium [7]. “Uninjured” respondents were those who had not suffered a sprain to either ankle. “Unstable” respondents were those who had reported a history of injury to either ankle greater than 1-year prior and scored above a 10 on the IdFAI questionnaire, indicating presence of CAI. “Coper” respondents were those who had reported a history of injury to either ankle greater than 1-year prior, but scored less than or equal to a 10 on the IdFAI indicating no presence of CAI with a history of ankle sprain. A fourth group of respondents included those reporting a history of ankle injury *within* the past year, and were therefore considered “potentially unstable” respondents as the International Ankle Consortium recommends a minimum of 1 year after injury to determine if coping or the formation of instability has occurred [7]. The FAAM-ADL and FAAM-Sport, as well as a composite score combining the two subscales were calculated as a percentage of perceived function for each participant. The IPAQ was scored using standard protocol with time in each activity to calculate PA for each participant as MET-minutes per week [22]. Finally, responses on recreational activities were tallied for frequencies within each group.

### Data analysis

To quantify the prevalence of ankle injury and CAI across the population, response frequencies were calculated to determine the proportions of individuals within each group for the entire cohort and then by age and sex. To determine differences in demographic characteristics and PA across groups, univariate analysis of variance (ANOVA) was used. Since data violated assumptions of parametric statistics, a Kruskall-Wallis test was used to assess difference across the groups. Next, categorical variables (age, recreational activities) were compared for frequency responses across groups using chi-squared analysis. Age was categorized into 1-year bins of age, ranging from 14 (youngest respondent) to 19 (oldest respondent). All analyses were conducted in IBM SPSS v 24.0 (IBM, Armonk, NY) and an a priori level of significance was set at 0.05. Interpretation of effect sizes were based on recommendations from Cohen [24].

## Results

Table 1 displays the demographic characteristics of the participants. The mean age of participants was 15.8 ± 1.2 years. Sixty percent of the participants were girls and 94% were white. The highest level of education that parents of participants have attained indicate that participants were from mostly well-educated families with over 70% having a college degree.

**Table 1:** Demographic characteristics of participants.

	*n*	%
Sex		
Boy	80	39.8
Girl	120	59.7
Did not disclose	1	0.5
Age		
14	33	20.2
15	38	23.3
16	46	28.2
17	36	22.1
18	8	5.0
19	2	1.2
Race/ethnicity		
Asian/Pacific Islander	7	3.5
Black/African American	4	2.0
Hispanic or Latino	9	4.5
Native American/American Indian	6	3.0
White/Caucasian	189	94.0
Other	2	1.0

Out of 201 participants, 115 (57.2%) reported a history of ankle sprain (boys 56.3%; girls 58.3%). Of these respondents, 40 (19.9%) reported a history of injury within the past year. Of participants more than a year from injury, 59 (78.6%) reported CAI (IdFAI ≥11), and 16 (21.3%) met the criteria of ankle copers (IdFAI≤10) (Table 2).

**Table 2:** Ankle injury groups.

Groups	*n* (%)	Age (yrs)	Height (m)	Weight (kg)	FAAM (*SD*)	FAAM-Sport (*SD*)	MET-minutes/week of physical activity (*SD*)^b^
Uninjured	86 (42.8)	15.98	1.69	61.94	89.54 (5.30)	87.42 (18.33)	2592.93 (2946.02)
Coper	16 (8.0)	15.38	1.66	65.24	90.00 (5.45)	89.84 (12.51)	3754.15 (3284.05)
Potentially unstable	40 (19.9)	15.63	1.65	60.86	86.38 (9.79)	88.29 (12.15)	3465.01 (3017.03)
Unstable	59 (29.4)	15.68	1.68	62.46	88.83 (8.23)	87.46 (13.85)	4706.05 (4610.56)^a^

^a^ Unstable participants reported more physical activity than uninjured participants (*p* < 0.01).

^b^Number of subjects completing a full IPAQ were: Uninjured, *n* = 71; Coper, *n* = 13; Potentially unstable, *n* = 35; Unstable, *n* = 46.

No significant differences were observed in total MET-minutes/week reported according to age (*F*_(5,157)_ = 1.49,*p* = 0.194) (Table 2). Chi-squared analysis revealed no significant differences in frequency of injury across ages $\left(\chi_{7}^{2}=0.18,p=0.27\right)$. A significant difference was observed, however, between sex and total MET-minutes/week reported (*t*(162) = 2.5,*p* = 0.036), with boys reporting more total MET-minutes/week than girls (boys = 4344.82 ± 4124.1 MET-minutes/week; girls = 2915.58 ± 3113.84 MET-minutes/week). Statistically significant differences were seen among the four ankle injury status groups for total MET-minutes/week, but the effect size was small $\left(\chi_{4}^{2}=11.65,p<0.01,\eta_{p}^{2}=.07\right)$. Whereas 201 cases were included for describing injury groups, the response rate for full IPAQ results was approximately 82% and similar across groups and age groups. Post hoc tests with a Bonferroni correction revealed unstable participants reported significantly more PA than uninjured participants (unstable = 4706.05 ± 4610.56 MET-minutes/week; uninjured = 2592.93 ± 2946.02 MET-minutes/week), with no differences between other groups (Table 2). No significant differences for main effect of injury status group were observed for ankle function using the FAAM $\left(F_{\left(3,145\right)}=1.62,p=0.19,\eta_{p}^{2}=.01\right)$ or FAAM-Sport $\left(F_{\left(3,145\right)}=0.12,p=0.87,\eta_{p}^{2}=.01\right)$. No differences in body mass index (BMI) were observed across groups (*F*_(3,185)_ = 0.41,*p* = 0.74). Groups were also not significantly different based on participation in high school sports $\left(\chi_{4}^{2}=4.38,p=0.22\right)$; however respondents with a history of ankle injury reported greater participation in jogging and running activities $\left(\chi_{3}^{2}=3.99,p=0.04\right)$; team sports $\left(\chi_{3}^{2}=8.37,p<0.01\right)$; and motor sports such as boating and riding motorcycles $\left(\chi_{3}^{2}=4.99,p=0.03\right)$.

## Discussion

The present study aimed to quantify prevalence rates of ankle injury among an adolescent population and to determine if differences existed in the type and amount of PA performed across groups with varying levels of ankle injury. Despite the younger age of our respondents, the prevalence rates of ankle injury in this rural adolescent population were similar to those previously reported in collegiate and general populations [12, 13]. When comparing PA levels and type, our findings refuted our *a priori* hypotheses as respondents with CAI participated in *more* self-reported PA than uninjured respondents. These data provide valuable insight as to potential risk factors for development of CAI after injury and probable effects on lifelong PA.

### Prevalence of injury

In the current study, 57% of adolescents reported a history of ankle injury and about 29% reported having CAI. Two previous investigations have attempted to highlight the prevalence of these common injuries among different populations. One study surveyed an adult population throughout Australia, reporting 60 percent of participants had a history of ankle injury and 24 percent had reported CAI [12]. Among an athletic population in American high school and collegiate settings, another study also reported over 60 percent of athletes had a history of ankle sprain and approximately 23 percent of athletes reported CAI [13]. Therefore, comparable rates of ankle injury history and CAI are seen among adolescents and adults as well as among athletes. The similar rates of ankle injury history may imply that adolescence is a high-risk age for sustaining ankle sprains. Due to the population this study evaluated, rural implications should also be considered. Rural adolescents self-report more major and minor injuries than their urban counterparts, however rural adolescents have a lower rate of medically treated injuries [25]. Research indicates that geographic distance and transportation to healthcare services may be a plausible explanation for the lower rates of seeking medical attention for injuries among rural populations [26, 27]. It is unclear as to why there is no difference in reporting of ankle injury between adolescents and adults as a reasonable expectation would be that adults report a higher prevalence of ankle injury than adolescents due to having more years of life and therefore, more chances to sustain an ankle injury. One potential hypothesis for this discrepancy is that PA levels tend to decline as age increases, therefore, decreasing the opportunities for exposure to injury with age [28]. The higher prevalence of CAI among adolescents reported in the current study (29 percent) compared to rates found in previous studies could also be attributed to the rural setting and participation in greater amounts of high risk physical activities [12, 13]. Longitudinal studies may attempt to understand the rates of ankle sprain and CAI over the lifespan to determine if age is associated with a decreased risk of injury.

No differences in ankle injury history were seen between boys and girls of the current study. This is contrary to a previous report from American emergency departments, where boys between the ages of 15–24 were observed to report with more ankle injuries than girls [11], although girls who are athletes in both high school and college have been reported to have higher rates of CAI than boys who are athletes [13]. The finding that boys of this study did not report a higher amount of ankle injury history is also surprising since boys consistently experience more injuries than girls [25]. However, among our cohort, boys engaged in more PA than girls as is consistent with other studies [3, 28–30]. A possible explanation for the difference in reported injuries could be that adolescent boys may underreport or not seek treatment for their ankle injuries compared to girls. For instance, another study reported that over half of their male adolescent participants did not seek medical attention for their ankle injury [31]. Furthermore, differences in frequency of injury by age was not seen in these data. The primary purpose of this analysis was to rule out the potential of a covariate in subsequent analyses; however, it is important to note that this dataset may have lacked sufficient power for truly assessing age differences, particularly in the upper age ranges.

### Physical activity in CAI

After categorizing the sample into four groups based on history of ankle injury and presence of instability, those that had CAI reported significantly more total MET minutes-per-week than the uninjured participants, indicating higher levels of PA. This finding is contrary to our a priori hypothesis and contradicts the results of a previous study conducted with a college age population [15]. This finding was independent of participant age, and no differences in BMI percentile-for-age and sex were noted. When considering responses regarding participation in recreational activities, respondents that had a history of ankle injury also reported more high-impact activities such as running, jogging, and team sports.

When combining these findings, it appears that among a rural adolescent population, ankle sprain is not a limiting factor for PA, and continued PA in high-risk sports may contribute to increased exposure that facilitates the development of CAI. A limiting factor in the diagnosis of CAI is that it relies on the primary symptom of “giving-way” in the ankle during activities of daily living. Subsequently, those who participate in regular PA and team sports may be more likely to experience the trademark symptom of CAI compared to uninjured populations, or those who may have discontinued PA after an injury and therefore may be able to “cope” with their injury. This may be a typical finding for adolescent populations as kinesiophobia, often described after CAI [8], may not affect younger populations. A study conducted on athletes at the high school and collegiate levels concluded that more than half of the sample reported no fear of returning to their sport after sustaining an injury that resulted in a loss of playing time (any injury, resulting in a loss of playing time not only ankle injuries) [32]. Subsequently, another study showed that pain was not a significant indicator of PA levels for adolescents [33].

Some caution must be taken with the interpretation of these findings as decreased PA levels have been described among collegiate-aged individuals [15, 34]. One of these studies utilized accelerometer-based technology to track step counts among a group of 20 patients with CAI and age-matched controls [15]. This is a notably different approach to the current investigation, as our study relied on self-reported PA through the IPAQ. One study comparing accelerometers and the IPAQ short form concluded that although the IPAQ short form has acceptable criterion validity, the IPAQ short form may over estimate self-reported PA compared to accelerometers [35]. While accelerometers may provide a potentially more accurate measure of PA, it is often difficult for them to obtain measures from a large cohort of individuals (as was attempted in this study).

Participants that have a history of ankle injury reported engaging in more high impact activities such as running, jogging, and team sports than the uninjured group. As such, we hypothesize that the reported high levels of PA may be leading to subsequent injuries specifically of the ankle and sensations of “giving way” or “rolling” of the ankle among those with injury history. Guddal et al. [36] also discovered that team sport participation was associated with an increased odds of lower extremity pain. Similar findings comparing athletes that engage in more strenuous activities versus patients who engaged in less strenuous activities reported a higher percentage of residual complaints such as CAI symptoms [37]. The current study found, however, that participants did not report decreased PA engagement in recreational activities despite injury. The most frequently reported recreational activities of the injured group were running, jogging, team sports and motor sports.

Limitations of this study include a small sample size and restriction of the population to a single rural high school population. These effectively limit the power of the study and the ability of the results to generalize the findings to a larger non-rural population. Recall bias due to self-reported PA levels may also have affected the amount of PA reported. As this study was cross-sectional, no determination regarding causality can be made. Furthermore, since data was collected during a time of the year when the weather in the study region was cooler, seasonality could have influenced participants’ overall PA and types of recreational activities. Future research should use longitudinal methods with larger sample sizes from both rural and urban settings to investigate if adolescents with high levels of PA and CAI reduce their PA levels over time.

## Conclusions

The findings of this study indicate that ankle injury rates among a rural adolescent population are similar to those reported among collegiate athletes and the general population, with nearly 60 percent of individuals having experienced an ankle sprain, and 30 percent reporting symptoms of CAI. Surprisingly, and contrary to previous reports, participants with CAI had higher levels of PA, indicating that this population may have further increased exposure to subsequent injury that elucidate the symptoms of CAI. Future research should prospectively investigate changes in PA levels after initial ankle injuries to determine how participation in subsequent PA increases or decreases risk of injury and subsequent instability in these populations and throughout age.

Ankle sprains have been highlighted as a potential public health crisis given high estimated annual costs from initial treatment alone, combined with long-term deficits in health-related quality of life. The results of this study suggest that these injuries significantly affect adolescent populations; however, affected individuals may be naturally more attracted to PA and therefore willing to risk reinjury. This potentially contributes to a negative feedback loop that can lead to long-term deficits in function. Sport medicine professionals should therefore consider targeting this population in an attempt to better ameliorate risk of reinjury, so that PA levels may not be affected later in life. Additionally, health education providers should consider educating this population on the negative and long-term impact of ankle injuries. Coaches and other health professionals should also encourage those that sustain an ankle injury to go seek medical attention or at least rehabilitate the injury appropriately. Without proper medical attention for an ankle injury, adolescents may experience long-term negative consequences to their ability to move and decreased participation in PA that can then lead to higher risk for chronic disease associated with sedentary lifestyles.

## Supporting information

S1 File Adolescent physical activity and CAI study data. This is the fully anonymized data set used for this study.
(SAV)

S2 File Chi-square analysis data. This is the data set used for chi-square analyses for this study.
(SAV)
